# Language impairments in children with developmental language disorder and children with high-functioning autism plus language impairment: Evidence from Chinese negative sentences

**DOI:** 10.3389/fpsyg.2022.926897

**Published:** 2022-09-28

**Authors:** Huilin Dai, Xiaowei He, Lijun Chen, Chan Yin

**Affiliations:** ^1^School of Foreign Languages, Shaoyang University, Shaoyang, China; ^2^Faculty of English Language and Culture, Guangdong University of Foreign Studies, Guangzhou, China; ^3^Center for Linguistics and Applied Linguistics, Guangdong University of Foreign Studies, Guangzhou, China; ^4^Department of Rehabilitation Medicine, The Central Hospital of Shaoyang, Shaoyang, China

**Keywords:** developmental language disorder, high-functioning autism plus language impairment, Chinese, negation, feature agreement

## Abstract

There is controversy as to whether children with developmental language disorder (DLD) and those with high-functioning autism plus language impairment (HFA-LI) share similar language profiles. This study investigated the similarities and differences in the production of Chinese negative sentences by children with DLD and children with HFA-LI to provide evidence relevant to this controversy. The results reflect a general resemblance between the two groups in their lower-than-TDA (typically developing age-matched) performance. Both groups encountered difficulties in using negative markers, which suggests that they might be impaired in feature agreement. Slight differences were detected between the two groups. Specifically, children with DLD experienced difficulties with the agreement on the feature [+telic] and that on the feature [+dynamic], while children with HFA-LI had difficulties with the agreement on the feature [+dynamic] and that on the feature [−dynamic]. This study supports the idea of a common symptomatology for the two disorders. More importantly, it suggests that these two disorders, DLD and HFA-LI, are not altogether the same in terms of language impairment. This paper concludes that general labels should not be simply attached to any children with language disorders. Instead, atypical language is very worthy of further analysis in the categorization of language disorders.

## Introduction

Developmental language disorder (DLD), also known as specific language impairment (Bishop et al., 2017), refers to a significant deficit in language ability that cannot be attributed to hearing loss, low nonverbal intelligence, or neurological damage (Leonard, 2014). Children with high-functioning autism (HFA) represent a subgroup of children with autism spectrum disorder (ASD). In the literature, individuals with HFA are described as having non-verbal intellectual functioning within the limits of typical development (Bishop, 2003; Gibson et al., 2013); some individuals with HFA have language impairment, whereas others do not. Children with DLD and those with HFA plus language impairment (HFA-LI) were reported as presenting similar symptoms in language, such as similar phonology (Demouy et al., 2011), vocabulary delay (McGregor et al., 2012), grammatical ability (Modyanova et al., 2017), and pragmatic errors (Kjelgaard and Tager-Flusberg, 2001; Norbury et al., 2013; van Santen et al., 2013). However, many studies have indicated different language or cognitive profiles between the two groups in lexicon tasks (Lloyd et al., 2006), grammar tasks (Schaeffer, 2018), non-word repetition tasks (Durrleman and Delage, 2016), and standard tests (Craig and Trauner, 2018). Some studies found that a general resemblance between the two clinical groups was observed in their lower-than-TD (Typically Developing) performance, but different error patterns were revealed in these two groups (Whitehouse et al., 2008; Riches et al., 2010; Sukenik and Friedmann, 2018; Chen et al., 2022). Reduced pragmatic abilities are the hallmark feature of ASD (Riches et al., 2010). Deficits in pragmatics also exist in children with DLD, although it is not the core feature (Roqueta and Katsos, 2020). These findings give rise to the controversy over whether the two groups share the same language impairments and whether their language disorders are caused by the same underlying deficits.

Agreement relationship is a locus of grammatical weakness in the DLD group (Clahsen et al., 1997; Tsimpli and Stavrakaki, 1999; Moscati et al., 2020). Deficits in feature agreement are striking characteristics of the DLD group’s difficulties with negative structures (Kunnari et al., 2014). Tests of negative structures also mirror the language impairments of the ASD population (Shapiro and Kapit, 1978; Tager-Flusberg et al., 1990; Schindele et al., 2008; Cuccio, 2012). Meanwhile, studies reveal that children with DLD show similar performance to younger TD children in negation (e.g., He and Dai, 2012; Thornton et al., 2016) as do children with HFA-LI (Shapiro and Kapit, 1978). Therefore, an analysis of the production of negative sentences may be useful in identifying the similarities or differences between the two groups.

Considering that children with DLD and children with HFA-LI show difficulties with the production of negation, and the fact that no study compared the two groups in the production of Chinese negative sentences, the present study investigates the production of Chinese negative sentences by children with DLD and those with HFA-LI to identify whether they demonstrate different impairments to help distinguish them clinically. Because early diagnosis and intervention can alleviate burdens on children’s cognitive resources and their language learning in school (Washington and Warr-Leeper, 2013), the participants in this study were preschool children.

### Feature agreement in Chinese negative sentences

In Chinese, *bu* “not” and *mei* “not” are the most frequently used negative markers and earliest acquired (Huang et al., 2022). In most cases, they are not interchangeable; otherwise, a negative sentence would be infelicitous to the corresponding affirmative statement. In a canonical Chinese negative structure like (1b), only the negative marker *mei* is appropriate to negate the affirmative statement in (1a), which indicates the achievement of an event *via* the aspectual marker *le*. Once the negative marker is substituted by *bu*, as in (2a), the negative sentence is ungrammatical or infelicitous in reply to (1a).


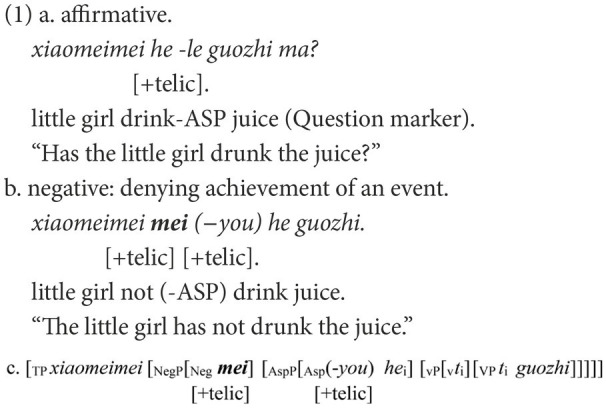



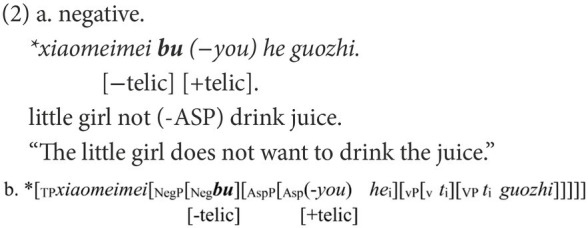


The ungrammaticality of (2a) is caused by the mismatch between the negative markers and the following functional head in the telic feature. A [+telic] feature has a natural final endpoint (Smith, 1991/1997). *Bu* has an inherent feature [−telic] whereas *mei* has an intrinsic feature [+telic], and they can occur only in situations compatible with these features (Li, 2007). In the affirmative sentence in (1a), the feature [+telic] is anchored in ASP *via* the aspectual marker *le*. In the corresponding negative sentence in (1b), via the covert *you* that has the same function as the aspectual mark *le* (Wang, 1965), the feature [+telic] is also embodied in ASP. When Neg is merged, it must agree with the *you* on the feature [+telic]. Only the negative marker *mei* with its feature [+telic] can fulfill the feature agreement, as in (1c). If *mei* is substituted by *bu*, the derivation will crash because of the failure of feature agreement between bu and you, as in (2b).

When *mei* is used to negate (1a), the negative sentence may be ungrammatical, as in (3a). The ungrammaticality can also be attributed to the failure of the feature agreement. A successful agreement can only be established between two elements. However, in (3), three elements are valid for agreement, and this is not allowed in the feature agreement. The derivation in (3b) is doomed to fail.


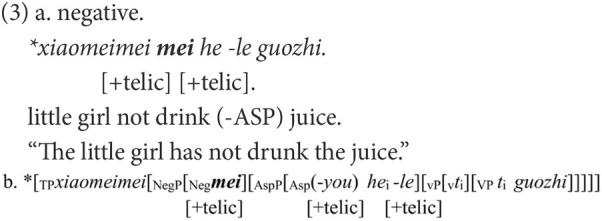


Another canonical pair of negative structures in Chinese concerns the negation of adjectives, as in (4b) and (5b). Despite the superficial resemblance in the linear order, their syntactic structures are different, as shown in (4c) and (5c). In (4), the functional head *a-become* introduces a change of states and embodies the feature [+dynamic] (Lin, 2004). The negative marker used to negate the affirmative statement in (4a) should also have a feature that is compatible with [+dynamic]. As the feature [+telic] entails the accomplishment of an event, the feature [+dynamic] can be deduced from [+telic]. In terms of dynamicity, the inherent features of *bu* and *mei* can be reinterpreted as the feature [−dynamic] being embodied in *bu* and the feature [+dynamic] in *mei*. Therefore, only *mei* can be the negative marker to negate (4a). Instead, if *mei* is replaced by *bu*, the agreement on feature [+dynamic] will fail, as in (4b). A similar situation is found in (5b), which demonstrates homogeneous states and embodies the feature [−dynamic].


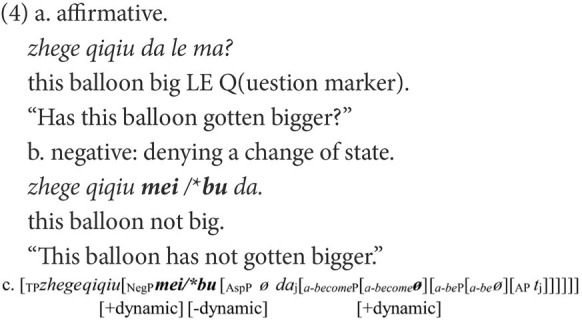



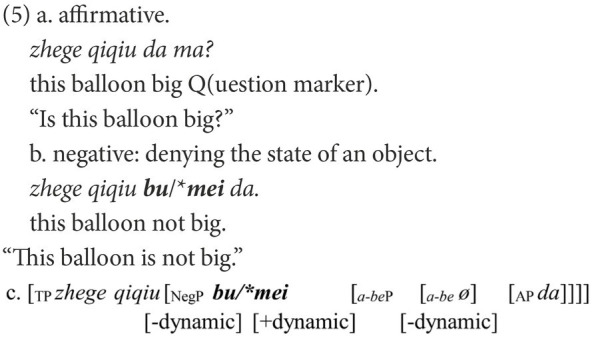


In Chinese, constituent omission is ubiquitous. The negative sentences in (1b), (4b), and (5b) are still grammatical when most elements are omitted, apart from the negative marker. Negative markers alone can completely fulfill their roles in denying the corresponding affirmative statement. Therefore, *via* Chinese negation, children’s knowledge of feature agreement can be inspected in a more straightforward manner.

### Negative sentences in children with DLD and children with HFA-LI

Children with DLD are claimed to perform similarly to younger TD children in an extended period of language development (Wexler, 1998). English-speaking children with DLD, like their younger TD peers, produce ungrammatical/non-adult negative sentences, such as *It not fit, It’s not fit, It not fits, It’s not fits* (Thornton et al., 2016). As expected, Chinese children with DLD have been also observed to perform similarly to younger TD children. Mistakes in using negative markers *bu* and *mei*, like (2a), (3a), (4b), and (5b), are typical of Chinese young TD children’s negative utterances (Zhang et al., 2006; Fan, 2007). Incorrect use of *bu* and *mei* was also observed in negative sentences of Chinese children with DLD. Tsai (2011) found that Chinese children with DLD were not sure about the different usages of *bu* and *mei*, and made a lot of mistakes in using these two negative markers, especially in negating sentences with an aspectual marker, such as (1a). He and Dai (2012) reported that Chinese children with DLD encountered severe difficulty in negating Chinese adjectives, like those in (4) and (5).

English children with ASD were also reported to interpret negative sentences in a manner comparable to their younger TD peers (Shapiro and Kapit, 1978). However, Shapiro and Kapit only reported the poor performance of children with ASD in the negative sentences. They did not specify the underlying clinical causes of the children’s difficulties. Until now, no published study has reported whether children with ASD have difficulty with the acquisition of Chinese negation, and no study has compared the production of negative sentences by Chinese children with DLD and those with ASD.

The failure in feature agreement is the underlying cause of ungrammaticality in some negative sentences as discussed in the previous section. The proposition that the agreement relationship is a locus of grammatical difficulty in children with DLD has been confirmed in several studies investigating children with DLD speaking English (Clahsen et al., 1997), Dutch (Blom et al., 2014), French (Paradis and Crago, 2001), German (Rice et al., 1997), Hebrew (Dromi et al., 1999), Italian (Rispens and Been, 2007), and Finnish (Kunnari et al., 2014). A question remains to be investigated is, whether children with ASD also have difficulty with feature agreement.

Although Chinese is a language without inflections as the languages studied previously, feature agreement does determine the grammaticality of language production. As discussed before, failure in feature agreement may result in the crash of the derivation of Chinese negative sentences, like (2a), (3a), (4b), and (5b). The Chinese negative markers *bu* and *mei*, valued differently in grammatical features, must fulfill feature agreement during the derivation of negative structures. The two negative markers can assume roles in negating on their own, without criticizing any other constituents. Thus, the hypothesis that children with DLD or those with HFA-LI are impaired in feature agreement will be more convincing if they are proved to have difficulty with Chinese negative structures.

### Research questions and predictions

As mentioned in the previous sections, Chinese negation could offer an effective linguistic means to compare the language of children with DLD and those with HFA-LI. The research questions are: What are the similarities or differences in the production of Chinese negative structures by children with DLD and those with HFA-LI? Are the underlying causes of the potential difficulties the same in the two groups?

Mandarin-speaking children with DLD are expected to perform similarly to younger TD children and produce ungrammatical negative sentences through the misuse of negative markers that are related to the failure in feature agreement. Meanwhile, children with ASD have performed similarly to younger TD children in negative sentences (Shapiro and Kapit, 1978). Therefore, Mandarin-speaking children with HFA-LI may also produce ungrammatical negative structures in the same way that younger TD children do. If ungrammatical negative sentences, such as (2a), (3a), (4b), and (5b), are detected in their production, children with HFA-LI may also be impaired in feature agreement. Thus, these two groups will be considered sharing the impairment in feature agreement. However, if none of the typical ungrammatical negative sentences are found in the HFA-LI group, these children may not have deficits in feature agreement. Then, these two groups may be impaired in different domains.

## Experiments

Two experiments were conducted to investigate the production of Chinese negative structures by children with DLD and those with HFA-LI, to see whether they display similar performance and to find out their similar and different underlying impairments in feature agreement.

### Experiment 1

Experiment 1 was designed to see whether children with DLD and children with HFA-LI have difficulties with the agreement on the feature [+telic] in Chinese negative structures. As discussed above, young children are likely to make mistakes in negating (1a), and this is relevant to failure in agreeing the feature [+telic] of the negative marker *mei*. Hence, the test sentences were the corresponding negative structure of (1a) in this experiment, namely, NP-*mei*-VP (Structure A).

#### Participants

Eighty-one preschool children were recruited and divided into three groups: the DLD group (*n* = 21, mean age = 62.6 months), the HFA-LI group (*n* = 32, mean age = 63.32 months), and the typically developing age-matched (TDA) group (*n* = 28, mean age = 62.6 months). All the children were from mainland China and spoke Mandarin Chinese. The children with language impairments were recruited from rehabilitation centers and kindergartens, and the TDA children were from kindergartens.

Before being included in the study, participants were required to undergo standard IQ and language competence tests. The *Wechsler Preschool and Primary Scale of Intelligence-Fourth Edition* [Chinese version; WPPSI-IV (CN)] was conducted to test the children’s non-verbal index (NVI; Li and Zhu, 2012). All participants in the present study had a NVI within the normal range. To be more specific, all scores of NVI are not lower than 73. The *Peabody Picture Vocabulary Test-Revised Chinese Version 1990* (PPVT-R) was used to test suspected children’s receptive vocabulary (Sang and Miao, 1990), and the *Rating Scale for Pre-school Children with Language Disorder-Revised Chinese* (RSPCLD-R; Lin et al., 2008) and the *Rating Scale for School Children with Language Disorder-Revised Chinese Version* (RSSCLD-R; Lin et al., 2009) were adopted to test the children’s language comprehension, production, and development. Four indexes were obtained for each child: PPVT-R, language comprehension (LC), language production (LP), and language development (LD). All the children with DLD and those with HFA-LI had at least two of the four indexes a minimum of 1.25 SD below the norms for their age (following Tomblin et al., 1997), and all the TDA children had scores above the cutoff for the normal range for their age in the four indexes (Table 1).

**Table 1:** Group details.

		DLD (*N* = 21)			HFA-LI (*N* = 32)			TDA (*N* = 28)		
		**Mean**	**SD**	**Range**	**Mean**	**SD**	**Range**	**Mean**	**SD**	**Range**
Age in months		62.6	6.22	52.93–76.24	63.32	7.82	44.52–77	62.6	5.22	55.2–77.62
PPVT-R		31.24	13.65	16–63	45.63	19.45	17–85	76.82	15.61	48–119
WPPSI-IV (CN)	VCI	83.81	6.42	69–96	87.5	11.35	71–114	106.93	8.05	90–126
	VSI	92.71	9.3	75–106	98.91	13.24	78–129	112.57	11.58	86–135
	FRI	92.38	13.61	69–130	101.97	13.46	72–133	107.07	8.94	86–123
	WMI	92.48	8.94	79–118	93.41	10.14	79–118	100.54	11.06	82–131
	PSI	95.52	8.08	75–109	91.91	10.25	73–109	106.43	8.41	86–124
	FSIQ	88.19	8.23	75–104	91.84	11.57	76–124	107.43	7.71	97–127
	NVI	92.05	9.57	73–108	97.78	11.82	81–128	107.57	9.25	91–132
	CPI	92.67	8.67	73–111	89.84	9.99	79–115	100.89	19.31	90–128
RSPCLD-R/ RSSCLD-R	LC	18.48	4.82	10–29	18	5.53	6–27	32.32	2.09	28–37
	LP	25.9	5.35	18–34	27.09	5.73	12–38	41.14	2.22	36–45
	LD	44.48	7.67	31–57	45.09	9.71	21–58	73.43	3.57	67–81

VCI, Verbal Comprehension Index; VSI, Visual Spatial Index; FRI, Fluid Reasoning Index; WMI, Working Memory Index; PSI, Processing Speed Index; NVI, Non-Verbal Index; CPI, Cognitive Proficiency Index; LC, language comprehension; LP, language production; LD, language development.

As confirmed by their parents and teachers, no child in the DLD or TDA groups had hearing abnormalities or a history of otitis media with effusion, neurological dysfunction, structural anomalies, oral-motor dysfunction, or any symptoms of impaired reciprocal social interaction. All children in the HFA-LI group had reports of an official diagnosis as ASD. Their ASD diagnosis had been confirmed by a pediatric neurologist based on the *Diagnostic and Statistical Manual of Mental Disorders* (DSM-5; American Psychiatric Association, 2013). All the parents signed consent for their children’s participation, which was approved by the Medical Ethics Committee of Xi’an TCM Hospital of Encephalopathy.

The three groups were matched on age [*F*(2, 78) = 0.117, *p* = 0.89]. The DLD and HFA-LI groups were matched on language ability. No significant difference was found between the DLD and HFA-LI groups in the Verbal Comprehension Index (VCI) of WPPSI-IV (CN; *p* = 0.468), which indicates children’s verbal ability (Ding et al., 2006; Zhang, 2009). Meanwhile, the DLD and HFA-LI groups were matched on full-scale IQ (*p* = 0.531), NVI (*p* = 0.161) of WPPSI-IV (CN) and on LP of RSPCLD-R/ RSSCLD-R (*p* = 1.000).

#### Materials and procedures

An elicited production task (Thornton et al., 2016; Schaeffer, 2018) was used to investigate the production of Structure A (NP-*mei*-VP). The participants were required to describe the actions indicated by the experimental materials.

Since negative statements are felicitous only when the corresponding affirmative propositions are considered (Wason, 1965), the felicity condition was satisfied in the present experiment by eliciting true affirmative statements, followed by those in which the affirmative proposition was false.


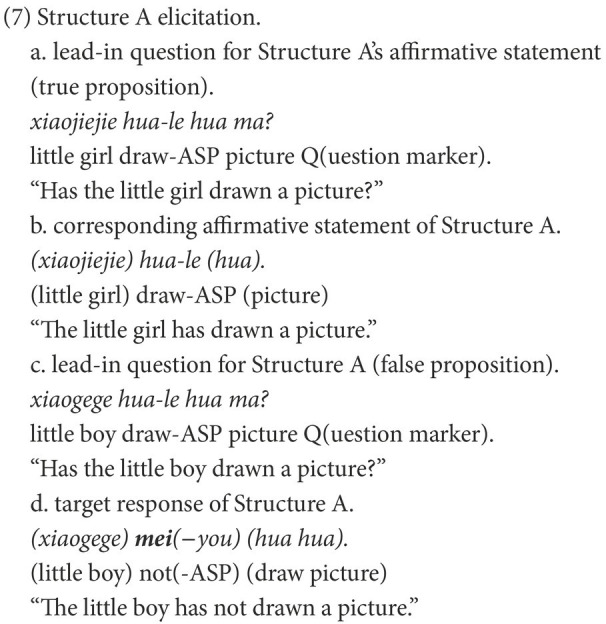


**Figure 1:**
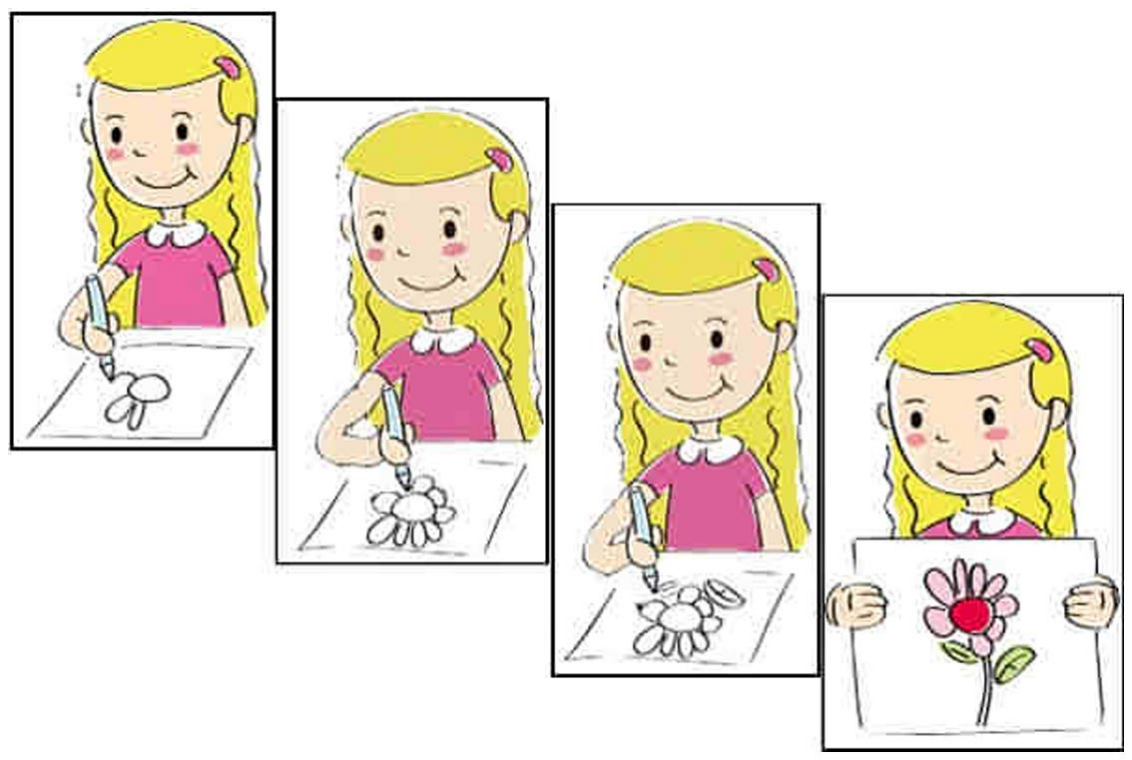
Pictures for (7a) and (7b).

**Figure 2:**
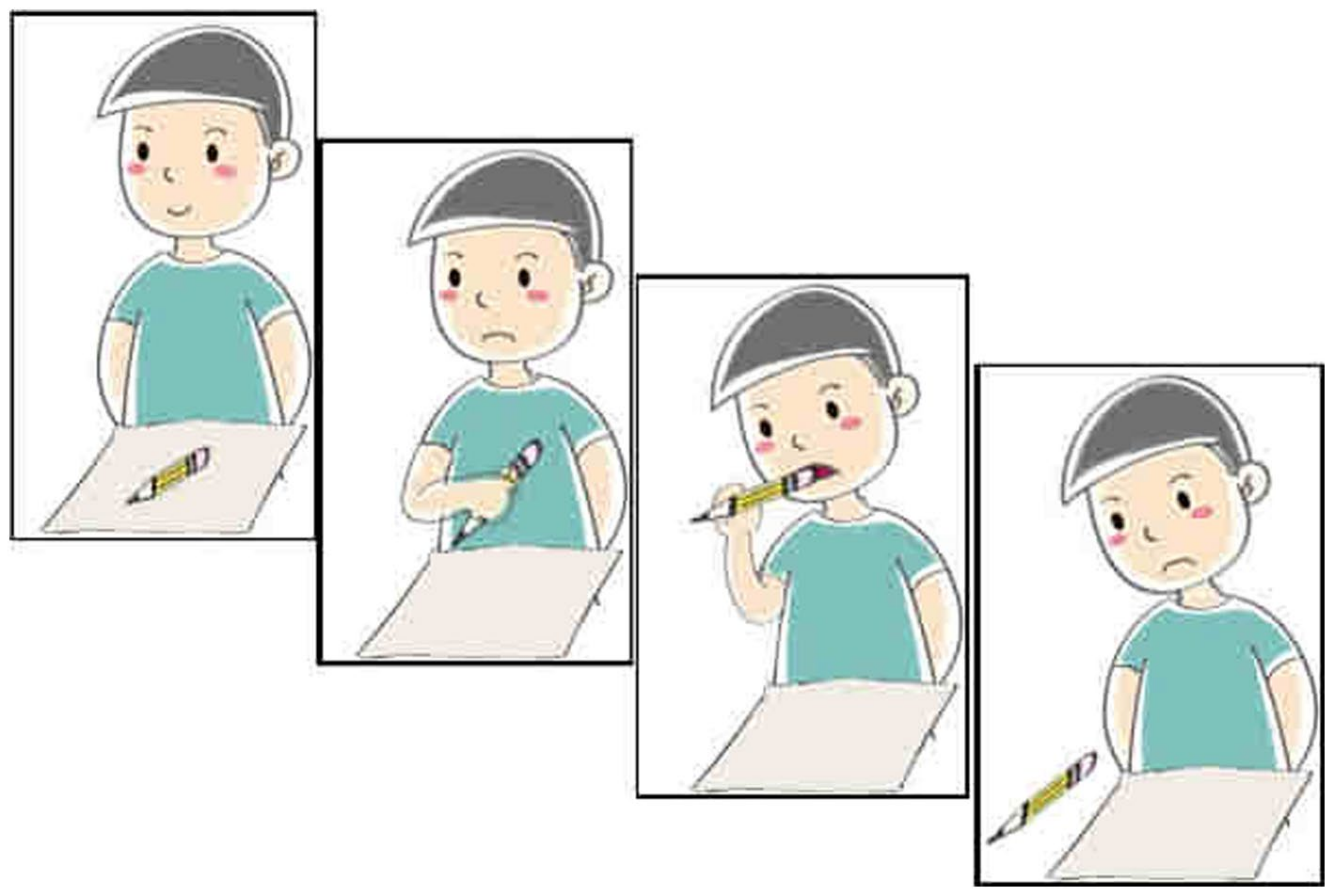
Pictures for (7c) and (7d).

A typical trial of Structure A is illustrated in (7). Four pictures were presented in superimposition to construct a GIF image, as shown in Figures 1, 2. This was done to create a felicitous context for the dynamic process involved in Structure A and its corresponding affirmative statement. Five trials were conducted, in which five verbs were used, including *chi* “eat,” *he* “drink,” *hua* “draw,” *da* “build,” *xizao* “have a bath.” In each trial, a lead-in question for a target negative sentence was presented only after the participant produced the corresponding affirmative statement. Two practice items that are similar to the experiment trials were provided for each participant to familiarize them with the task. All pictures were presented to the participants *via* Microsoft PowerPoint 2013.

#### Coding

The responses were classified into target and non-target. As subjects, objects, and even verbs can be omitted in Chinese negative sentences, a target response is the target negative sentence or an elliptical sentence that has the correct negative marker and expresses the correct semantic meaning, as in (8). Other forms of responses were marked as non-target, of which the affirmative sentences were categorized as Affirmative, as in (9), while the negative sentences were categorized as Negative. In the non-target negative responses of Structure A, some are grammatical and others are ungrammatical. The former were coded as Grammatical Negative, as in (10); the latter were coded as Ungrammatical Negative, as in (11).


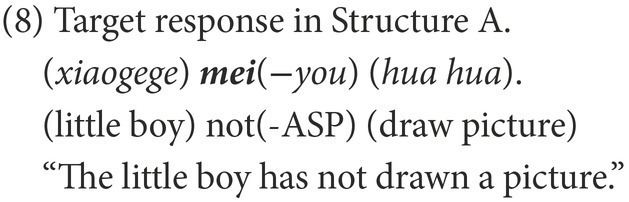



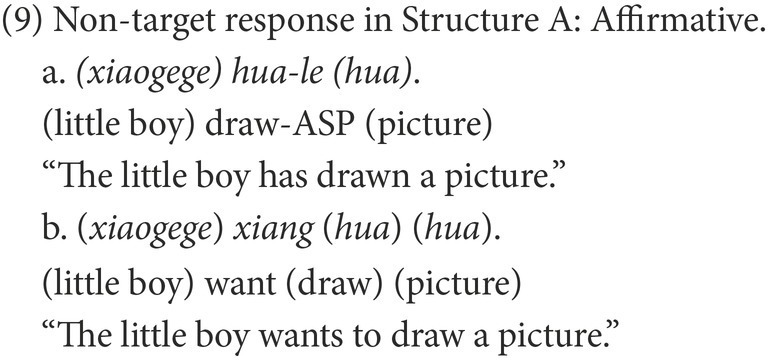



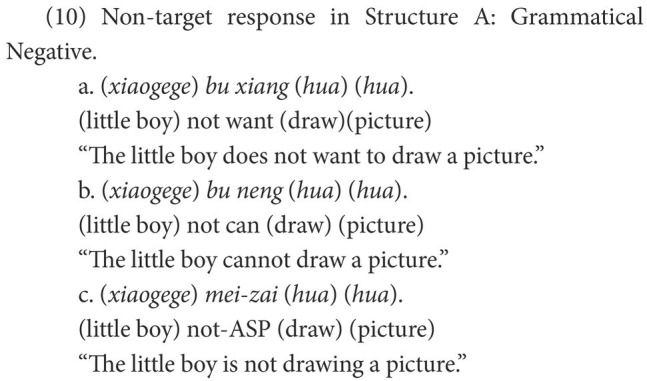



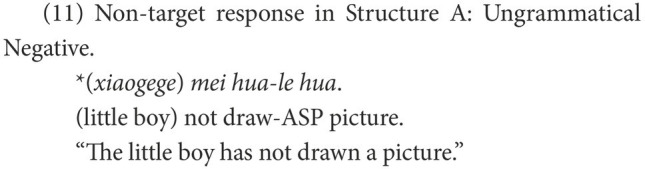


#### Results

Each child received all trials of the target negative sentences. The data collected in each task were analyzed using SPSS 24.0. Table 2 shows the descriptive statistics of the three groups in producing Structure A.

**Table 2:** Descriptive statistics of the three groups in Structure A.

	DLD	HFA-LI	TDA
Raw score (N)	77/105	83/160	139/140
Mean	3.67	2.59	4.96
SD	1.68	2.21	0.19

**Figure 3:**
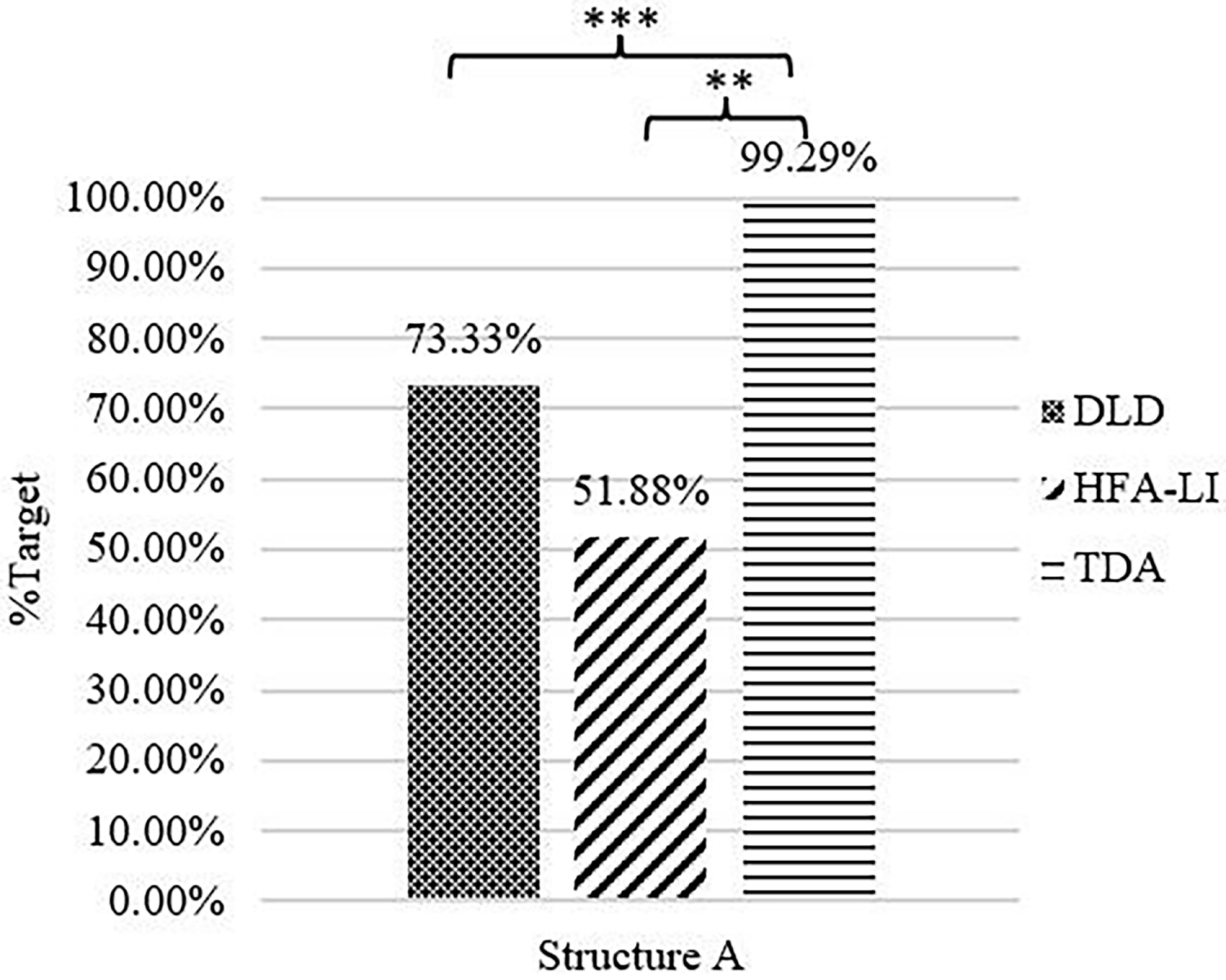
Percentage of target responses in Structure A. ** = significance (*p* < 0.01), *** = significance (*p* < 0.001).

Children in the DLD and HFA-LI groups did not perform well in the production of Structure A, whereas the TDA children reached the ceiling level (Figure 3).

A Kruskal-Wallis test revealed significant between-group differences in the number of Structure A’s target responses [H(2, 81) = 27.213, *p* < 0.001]. The DLD and HFA-LI groups were both outperformed by the TDA group (DLD *vs*. TDA: adjusted *p* = 0.002; HFA-LI *vs*. TDA: adjusted *p* < 0.001). There was no significant difference between the DLD and HFA-LI groups (adjusted *p* = 0.737); however, distinct patterns emerged in their non-target responses. As Table 3 shows, the grammatical non-target negative responses were produced by more than half of the children with HFA-LI, and the ungrammatical non-target negative sentences were only detected in the children with DLD.

**Table 3:** Number of non-target responses in Structure A.

	DLD		HFA-LI	
	Response (%)	Children (%)	Response (%)	Children (%)
Affirmative	5 (4.76%)	6 (28.57%)	12 (7.50%)	8 (25%)
Grammatical negative	18 (17.14%)	8 (38.10%)	65 (40.62%)	21 (65.63%)
Ungrammatical negative	5 (4.76%)	3 (14.29%)	0 (0%)	0 (0%)

#### Discussion

##### DLD group’s difficulty with Structure A

As English children with DLD produced ungrammatical/non-adult negative structures in the experiments in Thornton et al. (2016), the DLD group in the present study produced ungrammatical sentences in Structure A, like those in (12), typical Chinese ungrammatical negative sentences. These findings were consistent with those in Tsai (2011) and indicated that children with DLD are not sure about the usage of negative markers in negating sentences with the aspectual marker-le. The co-occurrence of the negative marker *mei* and the aspectual marker *le* results in the feature conflict between *mei*, *le*, and the covert marker-you.


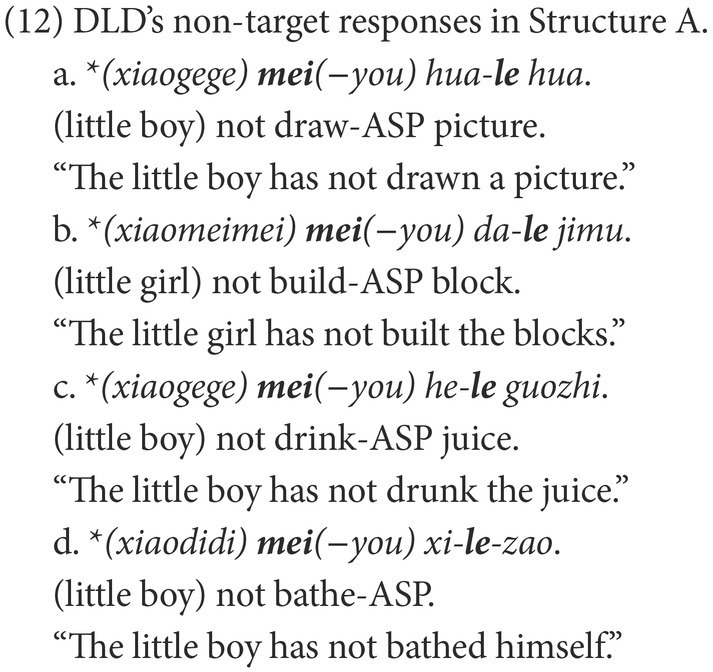


The incompatibility between *mei*, *le*, and *you* is relevant to the agreement on the feature [+telic]. The aspectual marker *le* and the covert marker *you* are in complementary distribution (Wang, 1965). The former is a suffix criticizing a verb in an affirmative sentence, while the latter occurs preceding a verb in a negative context. The covert *you* has a feature [+telic] that is anchored in ASP *via* the aspectual marker *le* in an affirmative sentence (Smith, 1994). When the negative marker *mei* is merged, the intrinsic feature [+telic] of *mei* must be checked.^1^ In feature agreement, a probe can only agree with the closest goal to check a matched feature. As shown in (13), the covert *you* is the closest goal to agree with *mei* on the feature [+telic].


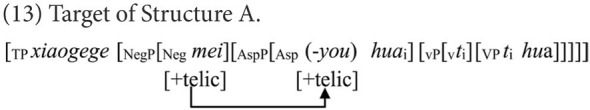



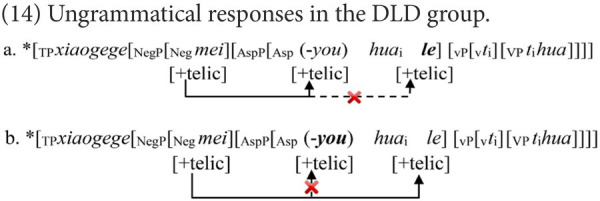


The ungrammatical negative sentences result from the failure to construct the agreement relationship of the feature [+telic]. In feature agreement, the probe and goal are in a biunique relationship. However, in the ungrammatical negative sentences of children with DLD, three elements, *mei*, *le*, and *you* need to be checked, preventing them from establishing the required relationship. When the negative marker *mei* is merged, if a child identifies *you* as the closest goal, the agreement between *mei* and *you* on the feature [+telic] leaves the feature of *le* unchecked; the derivation fails, as in (14a). If a child skips *you* and probes into *le*, the feature [+telic] is checked *via* the agreement between *mei* and *le*. Consequently, the derivation also fails because the feature of-you is not checked, as in (14b). Hence, we propose that Mandarin-speaking children with DLD are impaired in feature agreement.

##### HFA-LI group’s difficulty with Structure A

The HFA-LI group also encountered difficulties with Structure A, but the distribution of its non-target responses was not the same as that of the DLD group. Most non-target responses of the HFA-LI group are grammatical negative sentences, like those in (10), repeated in (15). Being irrelevant with agreeing any features, these responses cannot tell whether children with HFA-LI are impaired in feature agreement. However, they may provide another viewpoint.


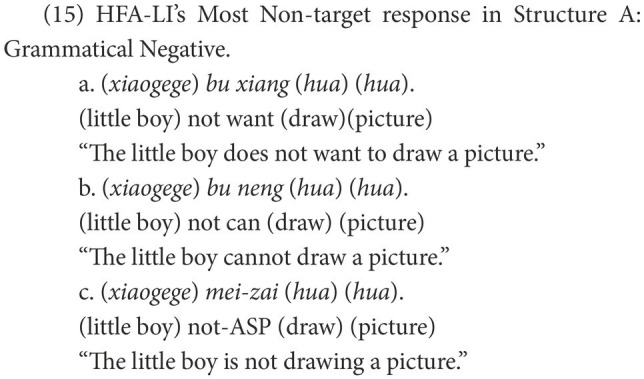


In Experiment 1, several pictures were presented in superimposition to construct a dynamic process of an event. To produce Structure A, each child must process and integrate the information between each picture/scene of a dynamic event, then decode and extract the general meaning of the negative statement. The sentences in (15) were infelicitous to reply to the lead-in question but matched one single scene/image during the dynamic process of the event. Probably affected by some deficits or impairments, children with HFA-LI were hindered from identifying the interconnection between the pictures/scenes, resulting in their failure to integrate the necessary information or attend to the global meaning. Instead, they may simply describe one picture/scene that they focused on. This must be a much easier strategy. Therefore, the superficial description of their focused scene(s)/image(s) yielded grammatical but infelicitous negative sentences in (15). Further studies are in need to find out what the underlying deficits or impairments prevent children with HFA-LI integrating scenes or generalizing information.

The experimental findings reveal that the DLD group and the HFA-LI group have difficulties with Structure A. The non-target responses suggest a minor difference between these two groups. Children with DLD may have difficulty in agreeing the feature [+telic], resulting in ungrammatical negative sentences. However, whether children with HFA-LI are impaired in feature agreement still remains an open question.

### Experiment 2

The previous experiment did not test whether the DLD and HFA-LI groups had difficulty in agreeing on features of the negative marker *bu*. Therefore, Experiment 2 was designed to investigate whether they performed differently in feature agreement. To be specific, Experiment 2 aims to find out whether these two groups have difficulties with the agreement on the feature [−dynamic] of the negative marker *bu* and on that of the feature [+dynamic] of the negative marker *mei*. A pair of negative structures relevant to adjectives was involved, i.e., NP-*bu*-adj. (Structure B) and NP-*mei*-adj. (Structure C).

#### Participants

The same three groups of children who participated in Experiment 1 also participated in Experiment 2, i.e., the DLD group (*n* = 21, mean age = 62.6 months), the HFA-LI group (*n* = 32, mean age = 63.32 months), and the TDA group (*n* = 28, mean age = 62.6 months). The three groups were matched on age and the DLD and HFA-LI groups were matched on language ability, full-scale, NVI and LP.

#### Materials and procedures

**Figure 4:**
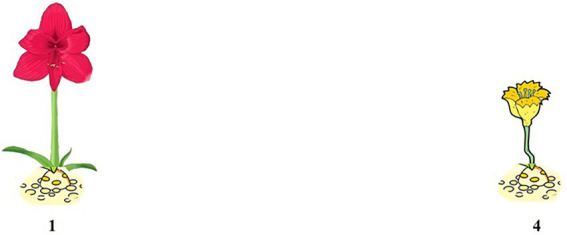
Last scene for (12e).

**Figure 5:**
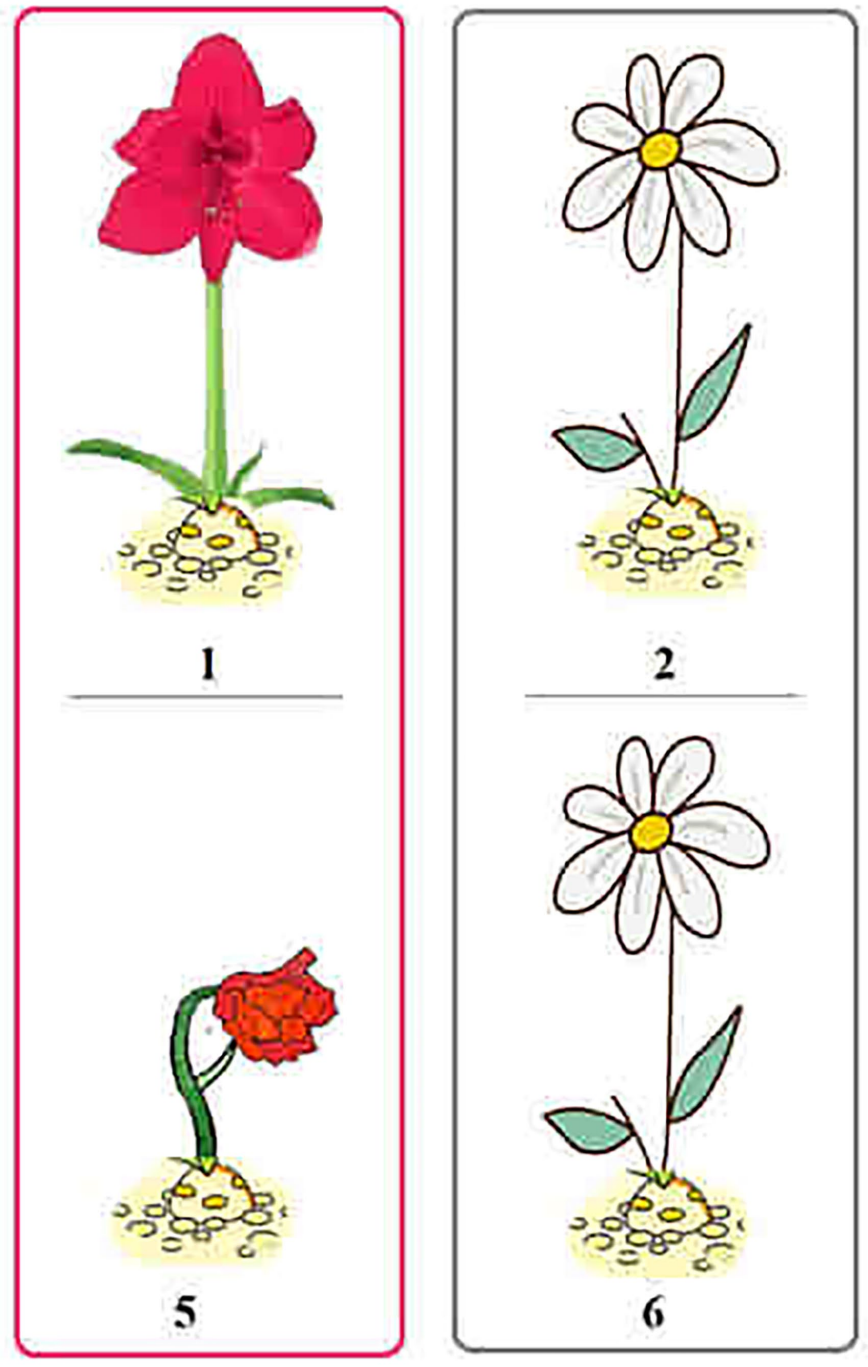
Last scene for (13e).


An elicited production task was adopted to investigate the production of Structure B (NP-*bu*-adj.) and Structure C (NP-*mei*-adj.). The target structures were elicited from a concise story. Typical trials are illustrated in (16) and (17). Figures 4, 5 show the last scenes of these trials.

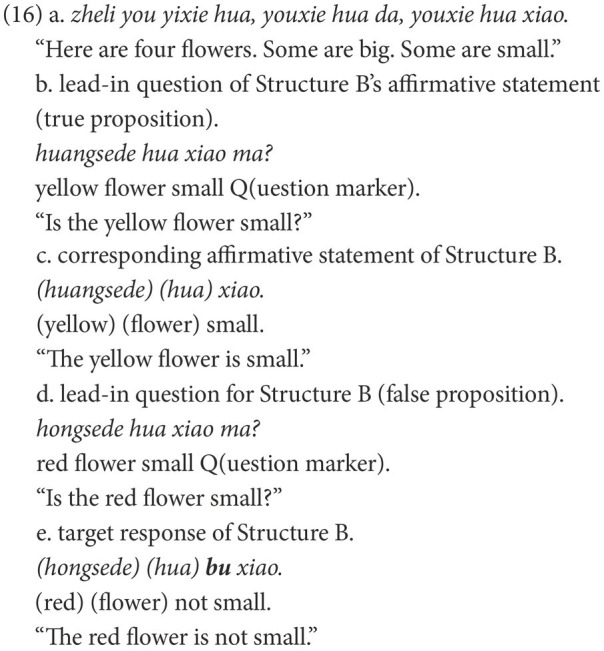



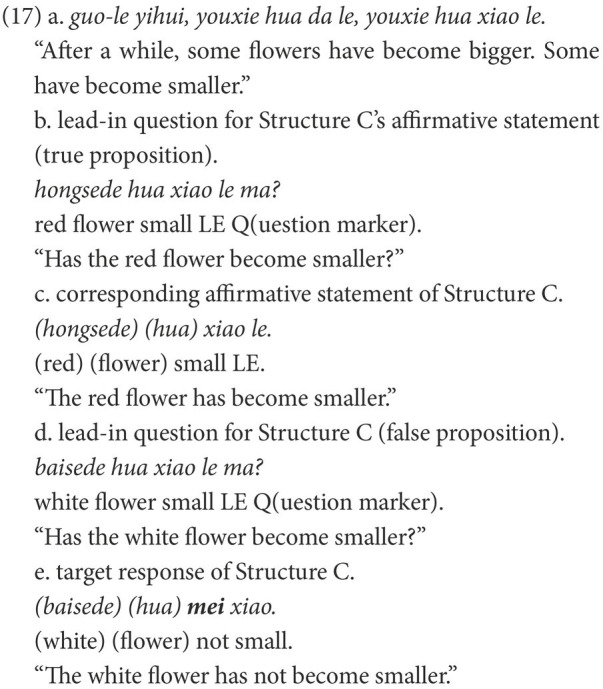


To avoid any by-effect from adjectives, pairs of an unmarked and a marked adjective, such as *da* “big” and *xiao* “small,” were included. For each structure, five pairs of trials were conducted. Ten adjectives were used, including *da* “big,” *xiao* “small,” *duo* “many,” *shao* “few,” *chang* “long,” *duan* “short,” *gao* “tall,” *ai* “short,” *zhong* “heavy,” *qing* “light.” As in Experiment 1, only after a child produced the corresponding affirmative statement, would he/she receive a lead-in question for a target negative sentence. Two practice items were provided for each participant. Microsoft PowerPoint 2013 was used to present all the materials to the children.

#### Coding

Participants’ responses were also categorized into target and non-target. A target response is the target negative sentence or an elliptical sentence with the correct negative marker, as in (18) and (21). Among the non-target responses, the affirmative sentences were coded as Affirmative, as in (19) and (22), while the negative sentences were coded as Negative, as in (20) and (23).


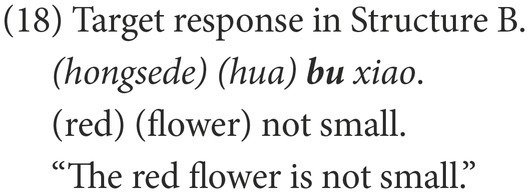



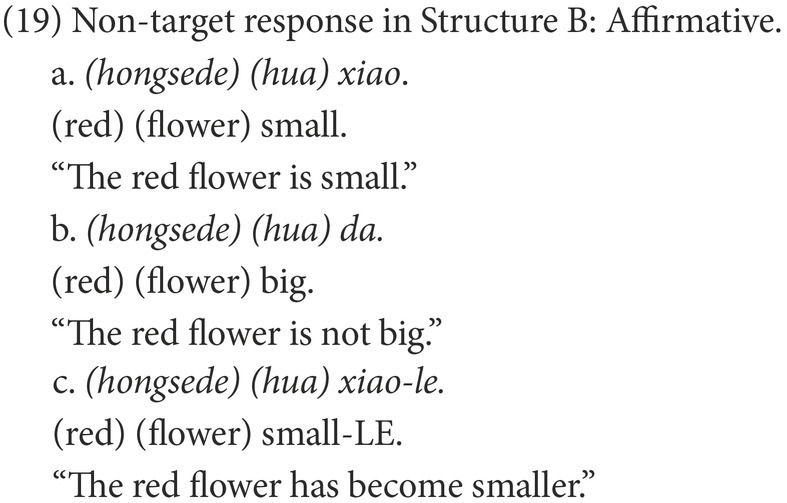



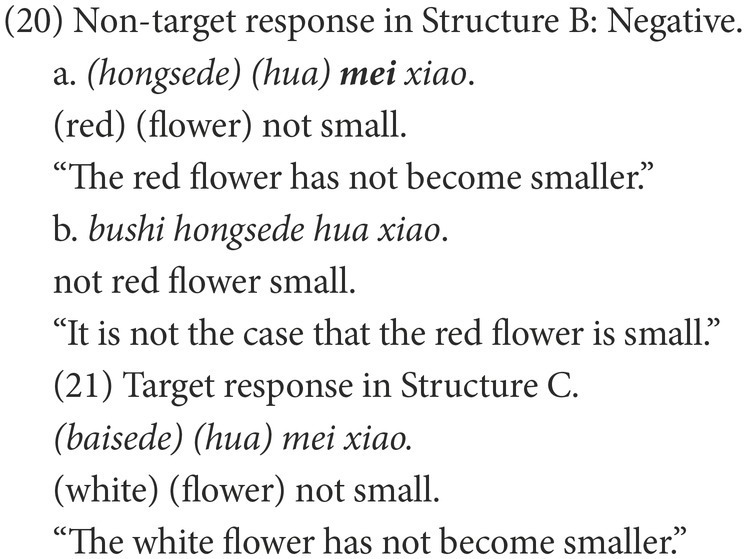



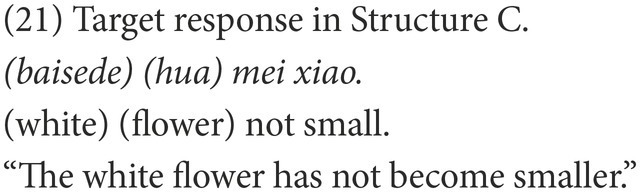



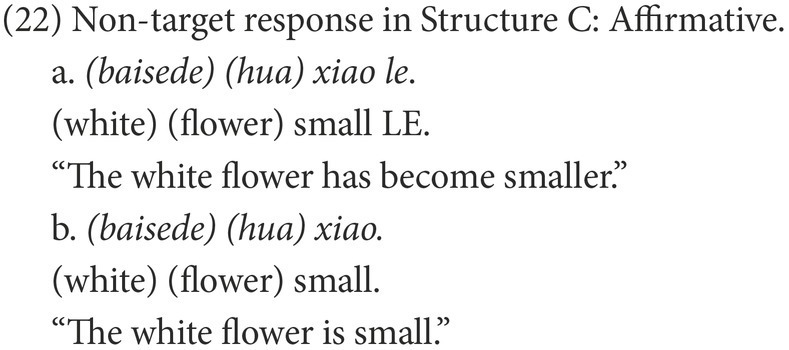



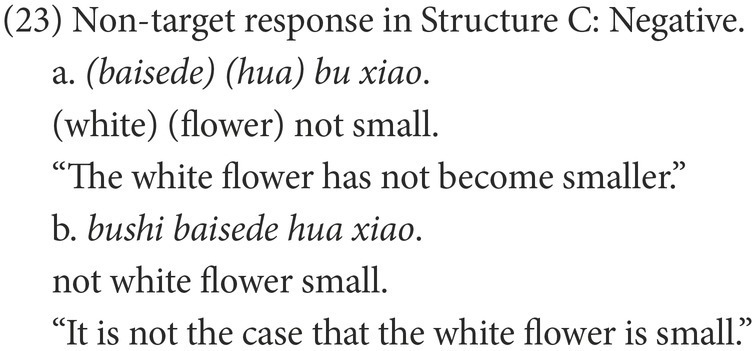


#### Results

Each participant received all trials of the target negative sentences in Experiment 2. The data collected in each task were analyzed using SPSS 24.0. Table 4 shows the descriptive statistics for the three groups in the production of Structures B and C.

**Table 4:** Descriptive statistics of the three groups in Structures B and C.

	Structure B			Structure C		
	DLD	HFA-LI	TDA	DLD	HFA-LI	TDA
Raw score (N)	193/210	202/320	273/280	107/210	158/320	253/280
Mean	9.19	6.31	9.75	5.1	4.94	9.04
SD	1.4	4	0.65	3.52	3.95	1.93

**Figure 6:**
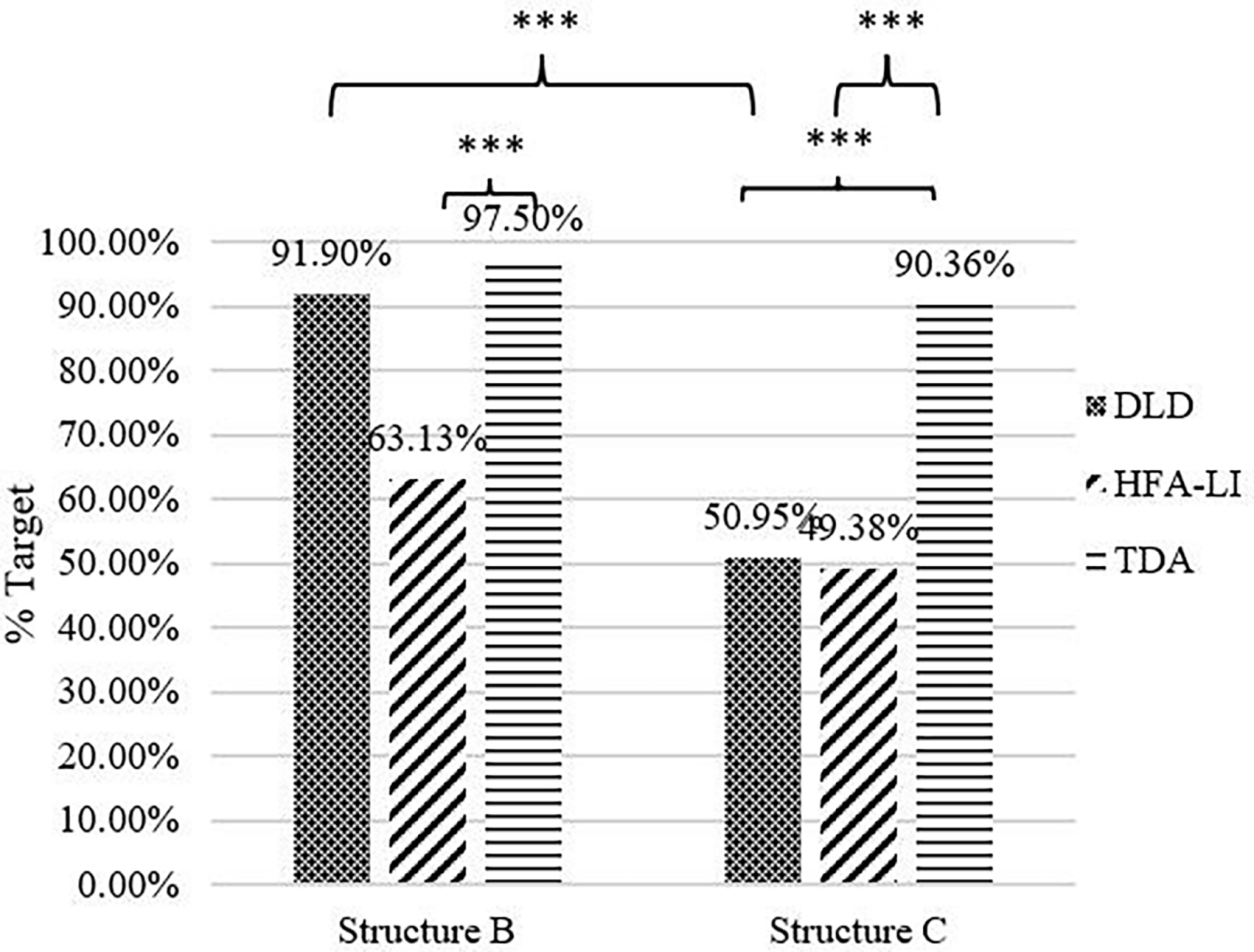
Percentage of target responses in Structures B and C. *** = significance (*p* < 0.001).

The results in Structure C displayed a similar pattern as in Structure A, but those in Structure B did not (Figure 6). Kruskal-Wallis tests indicated significant between-group differences in the number of target responses [Structure B: H(2, 81) = 17.778, *p* < 0.001; Structure C: H(2, 81) = 23.146, *p* < 0.001]. The pairwise comparisons showed that the HFA-LI group, but not the DLD group, was outperformed by the TDA group in Structure B (DLD *vs*. TDA: adjusted *p* = 0.423; HFA-LI *vs*. TDA: adjusted *p* < 0.001). In Structure C, the DLD and HFA-LI groups presented poorer performance than the TDA group (DLD *vs*. TDA: adjusted *p* < 0.001; HFA-LI *vs*. TDA: adjusted *p* < 0.001).

The difference between the DLD and HFA-LI groups was marginally significant in Structure B (adjusted *p* = 0.059), but not significant in Structure C (adjusted *p* = 1.000). However, the patterns of non-target responses are not identical in the two groups in Structure C (Table 5). The most frequent non-target responses in the DLD group were the negative ones, similar to those in (23). A substantial proportion of the children with DLD produced these non-target responses. In the HFA-LI group, the affirmative sentences were a little more than the negative ones, and those who produced these two categories of non-target responses were nearly the same in number and proportion.

**Table 5:** Number of non-target responses in Structure C.

	DLD		HFA-LI	
	Response (%)	Children (%)	Response (%)	Children (%)
Affirmative	22 (10.48%)	11 (52.38%)	82 (25.62%)	17 (53.13%)
Negative	81 (38.57%)	18 (85.71%)	62 (19.38%)	18 (56.25%)
Others	0 (0%)	0 (0%)	18 (5.62%)	10 (31.25%)

The two groups also showed different patterns among the non-target responses in Structure B (Table 6). In the negative responses, those sentences including the incorrect negative marker *mei*, such as (20a) were only observed in the HFA-LI group. In the affirmative responses, the sentences involving-LE, such as (19c) were only found in the HFA-LI group. Nearly one-third of the HFA-LI group, much more than the DLD group, produced non-target affirmative sentences, such as (19b), which are grammatical and have a similar meaning to the target negative responses.

**Table 6:** Number of non-target responses in Structure B.

	DLD		HFA-LI	
	Response (%)	Children (%)	Response (%)	Children (%)
Affirmative				
(19a)	9 (4.29%)	4 (19.05%)	32 (10.00%)	9 (28.13%)
(19b)	4 (1.90%)	4 (19.05%)	30 (9.38%)	10 (31.25%)
(19c)	0 (0%)	0 (0%)	15 (4.69%)	3 (9.38%)
Negative				
(20a)	0 (0%)	0 (0%)	27 (8.44%)	9 (28.13%)
(20c)	1 (0.48%)	1 (4.76%)	13 (4.06%)	4 (12.50)
Others	3 (1.90%)	3 (14.29%)	1 (0.31%)	1 (3.13%)

Distinct patterns, which we call stereotyped patterns, emerged in the HFA-LI group’s responses if a non-target response, its prior response, and its following response were all considered. A participant’s responses are categorized as a stereotyped pattern when three consecutive responses to the negative questions are in the same form. Four stereotyped patterns have been found. As (24) shows, in Type 1 and Type 2, the responses were copies of the last constituents in the lead-in questions. In Type 3 and Type 4, the negative marker *bu* or *mei* was repeatedly positioned before the adjectives.


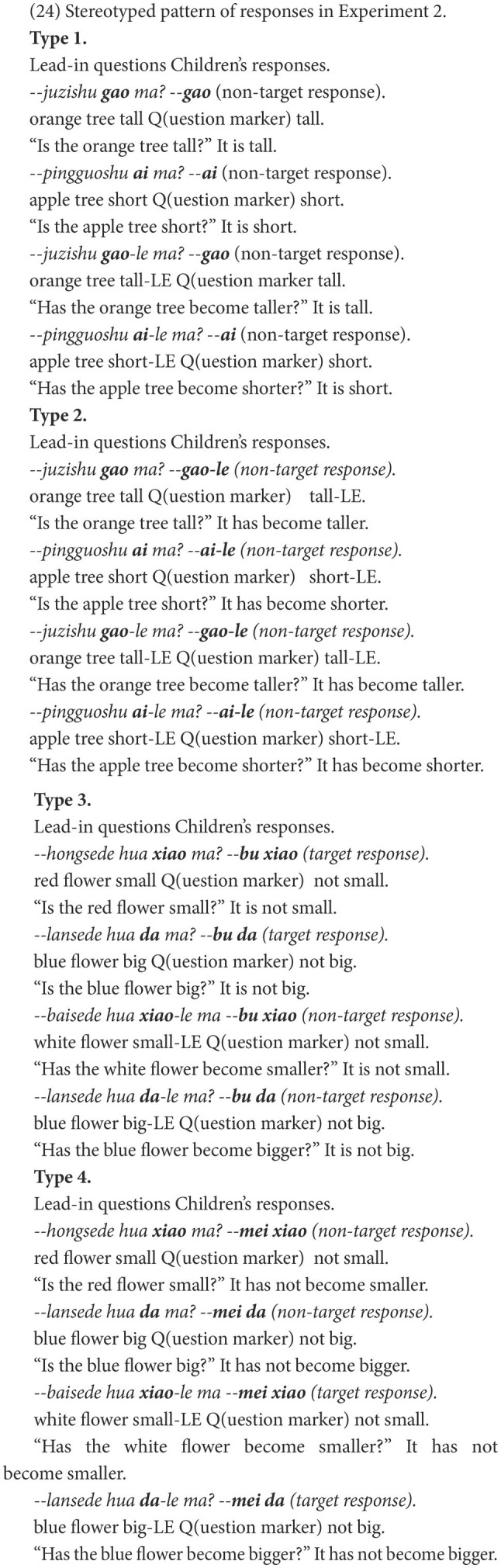


The HFA-LI group presented all four types of stereotyped responses, while the DLD group only produced the third type (Table 7). The children with HFA-LI were quite stereotyped in constructing the superficially resembled structures, especially in repeating negative markers. However, significantly fewer of the children with DLD presented the stereotyped responses.

**Table 7:** Number (N) and percentage (%) of children in stereotyped responses.

	DLD		HFA-LI	
	N	%	N	%
Type 1	0	0%	5	15.63%
Type 2	0	0%	3	9.38%
Type 3	8	38.1%	17	53.13%
Type 4	0	0%	6	18.75%

In short, the findings in Experiment 2 showed different performance patterns between the two clinical groups. The HFA-LI group presented poor performance in Structure B (NP-*bu*-adj.) and C (NP-*mei*-adj.), while this was only true of the DLD group in Structure C. Moreover, the two groups displayed different patterns in their non-target responses. Specifically, the DLD group substituted the negative marker *mei* with *bu* in substantial numbers of non-target responses (Table 5) but did not substitute *bu* with *mei* (Table 6). The HFA-LI group not only replaced *mei* with *bu*, but also *bu* with *mei*. The HFA-LI group was more likely to produce affirmative sentences with the target meaning (Table 6), and displayed stereotyped behavior in language production, but the DLD group did not (Table 7).

#### Discussion

##### DLD group’s difficulty with Structure C

The results in the DLD group’s production of Structure C were consistent with the findings in He and Dai (2012) that children with DLD had difficulties in using *mei* to negate adjectives. The DLD group’s poor performance in Structure C supports the conclusion in Experiment 1 that children with DLD might be impaired in feature agreement. Children with DLD substituted the negative marker *mei* with *bu* in a large number of non-target negative sentences in Structure C, such as (25b). During the derivation of Structure C, as in (25a), the negative marker must agree with *a-become* in the feature [+dynamic]. Therefore, the head of NegP can only be spelled out as *mei*, of which the feature [+dynamic] is a part.


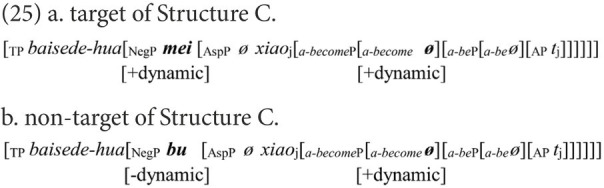


If a child has mastered the agreement on the feature [+dynamic], the correct negative marker *mei* will be yielded. However, as predicted, an incorrect negative marker was produced by children with DLD. They must be impaired in the feature agreement on the [+dynamic]. However, the substitution is asymmetric. *Bu* was not replaced by *mei* in the DLD group’s responses (Table 6). Therefore, the agreement on feature [−dynamic] is not a significant challenge to the DLD group.

Based on the DLD group’s difficulty with Structures A and C, we propose that Chinese children with DLD have deficits in the agreement on the features [+telic] and [+dynamic] of *mei* within their grammar system. This proposal is consistent with findings in other studies. Children with DLD speaking inflection-rich languages show enormous difficulties in morphosyntactic agreement on features (Clahsen et al., 1997; Moscati et al., 2020). Therefore, impairments in agreement relation seem to be ubiquitous among children with DLD, and feature agreement appears to be a locus of their difficulties.

##### HFA-LI group’s difficulty with Structures B and C

First, the HFA-LI group, rather than the DLD group, displayed difficulties in Structure B (NP-*bu*-adj.). The non-target negative responses, such as (20a) repeated here as (26b), suggest that children with HFA-LI are impaired in feature agreement.


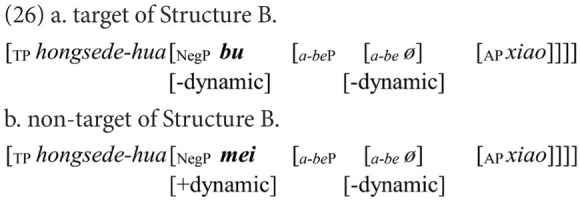


The verbalizing head, *a-be*, licenses static situations, embodying the feature [−dynamic] (Lin, 2004). In terms of feature agreement, the negative marker used in Structure B should also have the feature [−dynamic]. Therefore, *bu* equipped with the feature [−dynamic] is the proper negative marker. If the negative marker was *mei*, the derivation of Structure B would crash because of the failure in agreement between the feature [−dynamic] of *a-be* and the feature [+dynamic] of *mei*.

Such sentences failing in the agreement on the feature [−dynamic] account for a significant proportion of the non-target responses in the HFA-LI group, and a fair number of the children with HFA-LI made such mistakes. Hence, we propose that Mandarin-speaking children with HFA-LI are impaired in feature agreement.

This proposal is supported by the HFA-LI group’s performance in Structure C. In the derivation of Structure C, as shown in (25a), the feature [+dynamic] of the functional head *a-become* should be checked *via* agreement with the same feature of a negative marker. As (25) shows, the negative marker *mei* amply fulfills the feature agreement, but *bu* with its feature [−dynamic] is not appropriate for the agreement on the feature [+dynamic]. The HFA-LI group produced many non-target negative sentences, such as (25b), and quite a few of the children with HFA-LI made this mistake. These non-target negative responses in Structure C indicate that children with HFA-LI are also impaired in the agreement of the feature [+dynamic]. Therefore, the conclusion is more convincing that Mandarin-speaking children with HFA-LI are impaired in feature agreement.

Second, another significant finding is that the HFA-LI group produced affirmative sentences that are synonymous with the target negative ones like (27), but the DLD group did not. It is probable that the preference for an affirmative sentence is relevant to the processing load.


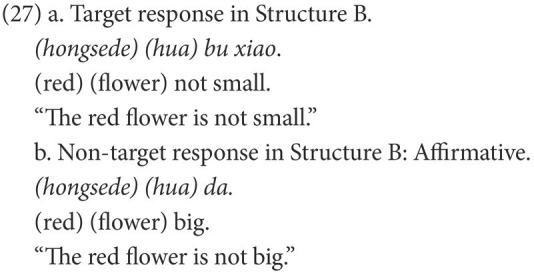


The plausibility of a negative sentence is closely connected to the presence of its corresponding affirmative statement (Wason, 1965; Horn, 1989). Based on this consideration, Kaup et al. (2005, 2006, 2007) propose the Two-Step Simulation Hypothesis, suggesting that a negative sentence entails a presupposition of its corresponding affirmative proposition, which needs to be corrected and then conveys the corrected information to a comprehender. Hence, in terms of mental presentation, two stages are constructed in sequence during the interpretation of a negative sentence. In the first stage, a comprehender represents the simulation of “the negated state of affairs” that corresponds to the negated situation. In the second stage, they construct “the actual state of affairs,” which matches the actual meaning of the negative sentence. As in (27a), a child should first construct the mental representation of the negated state of affairs, i.e., a small red flower, and then shifts to simulation of the actual state of affairs, i.e., a big red flower. If the affirmative sentence with the antonymous adjective, such as (27b), is taken into interpretation, only the actual state of affairs needs to be constructed. The processing load must be heavier in a negative sentence. In other words, a negative sentence is more difficult to interpret because its processing costs are enhanced. A substantial body of empirical works supports this hypothesis (Kaup et al., 2005, 2006, 2007; Hasson and Glucksberg, 2006). The fact that only the HFA-LI group presented such performance demonstrates that the extra cost of processing a negative sentence overburdens children with HFA-LI. However, it does not impose overload on children with DLD. The HFA-LI group may be more impaired in processing ability.

Third, as shown in (24) and Table 7, the HFA-LI group’s responses were stereotyped in Structures B and C, if a non-target response, its prior response, and its following response were all considered. Children with HFA-LI were apt to repeat some elements in the lead-in questions. To be specific, in the non-target responses, they copied adjectives in Type 1 and 2 and repeated negative markers in Type 3 and 4. This might be the phenomena of repetitive behaviors in language of children with HFA-LI that is a developmental disorder characterized by restricted and repetitive behaviors (American Psychiatric Association, 2013). Stereotyped language was not observed in the DLD group, indicating that children with DLD may not be affected by restricted and repetitive behaviors. However, this is just a preliminary inference. Further studies are in need to confirm whether the stereotyped language in the production of negative sentences or difficulties with the production of negative sentences are relevant to the HFA-LI group’s repetitive behaviors.

## Limitations

This work presented several limitations, two of which are acknowledged here.

First, the narrow age range of children and a limited number of participants may broaden individual differences in verbal and non-verbal ability. Although the DLD and HFA-LI groups were matched on language ability and on FSIQ and NVI of WPPSI-IV (CN), the ranges of FSIQ, NVI and VCI are a little bit wide, which may affect the results within or between groups. In the present study, such effects may not be significant because those children in the DLD or HFA-LI group who got high scores in NVI or VCI did not perform well in the production of negation.

**Figure 7:**
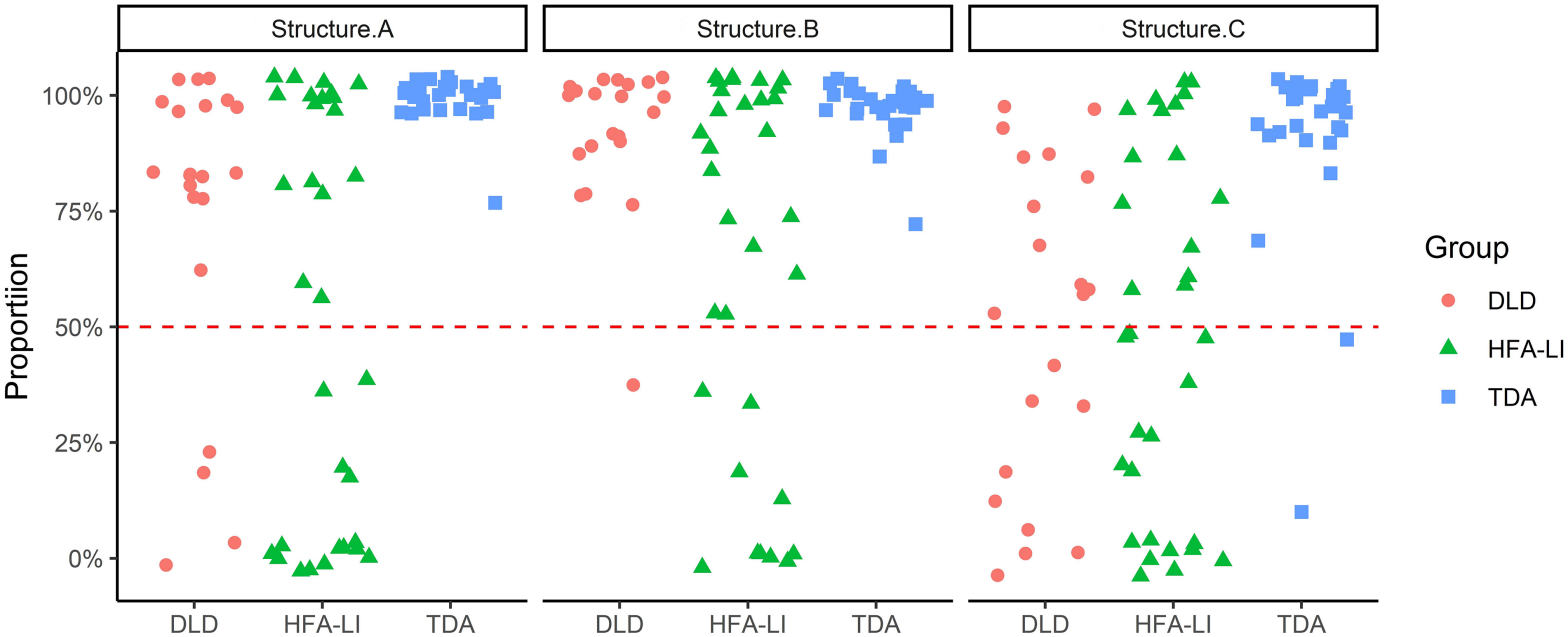
Each participant’s performance on each structure.

The performance of each individual on the production of the three structures is shown in Figure 7. When we consider the performance of the DLD and HFA-LI groups, no one in the two groups did extremely well or poorly in the production of all the three structures. Those who performed relatively worse got medium scores in NVI and VCI of WPPSI-IV (CN).

Second, as the previous studies indicated that pragmatic impairment is the hallmark feature of ASD (Riches et al., 2010) and deficit in pragmatics also exists in children with DLD (Roqueta and Katsos, 2020), the present study did not exclude the pragmatic impairment of the participants.

Future studies should enlarge the participant sample and pay more attention to individual differences and participants’ pragmatic impairments. Such work will be of great significance to a comprehensive understanding of the development of children’s knowledge of negation.

## Conclusion

Similarities and differences in language impairments were found between the DLD and HFA-LI groups in their lower-than-TDA performances in the production of Chinese negative structures. The impaired feature-agreement relation in the grammatical system might be the main cause of the DLD group’s difficulty. Similar impairments in feature agreement are also characteristic of the HFA-LI group.

A slight difference between their impairments in feature agreement lies in the specific features in which these two groups of children encountered difficulties. The ungrammatical negative sentences and asymmetrical substitution of *bu* for *mei* in the DLD group suggest children with DLD experienced difficulties with the agreement on the feature [+telic] and that on the feature [+dynamic], while the replacement between *bu* and *mei* in the HFA-LI group indicates children with HFA-LI had difficulties with the agreement on the feature [+dynamic] and that on the feature [−dynamic]. Meanwhile, the DLD group may have a higher tolerance to the processing load, but lower tolerance in processing burden might intensify the HFA-LI group’s hardship in the production of negative sentences.

The findings in the present study further support that DLD and HFA-LI share a common symptomatology (Georgiou and Spanoudis, 2021), and indicate that these two disorders are not altogether the same. Based on the findings, we propose that one child with language impairments cannot be simply labeled as having any one disorder. However, atypical language needs further analysis for confirmation of language disorders (Sukenik and Friedmann, 2018; Chen et al., 2022).

## Data availability statement

The raw data supporting the conclusions of this article will be made available by the authors, without undue reservation.

## Ethics statement

The studies involving human participants were reviewed and approved by Medical Ethics Committee of Xi’an TCM Hospital of Encephalopathy. Written informed consent to participate in this study was provided by the participants’ legal guardian/next of kin.

## Author contributions

HD and XH conceived the study. HD carried out the experiments and draft the manuscript. HD and CY analyzed and interpreted the data. LC and CY contributed to the revision of the body part of the manuscript. All authors contributed to the article and approved the submitted version.

## Funding

This research was supported by the National Social Science Fund of China (no. 17AYY008) and by Philosophy and Social Science Foundation of Hunan Province (no. 19YBQ094).

## Conflict of interest

The authors declare that the research was conducted in the absence of any commercial or financial relationships that could be construed as a potential conflict of interest.

## Publisher’s note

All claims expressed in this article are solely those of the authors and do not necessarily represent those of their affiliated organizations, or those of the publisher, the editors and the reviewers. Any product that may be evaluated in this article, or claim that may be made by its manufacturer, is not guaranteed or endorsed by the publisher.

## Footnotes

^1^In this paper, we propose that the features checked *via* agreement should be the same in value.
